# Ultrasound identification of absent rectus abdominis muscle after omphalocele repair altered regional anesthesia strategy in pediatric cardiac surgery: a case report

**DOI:** 10.1186/s40981-026-00863-7

**Published:** 2026-05-25

**Authors:** Tomohiro Yamamoto, Teppei Yamada, Shuichi Shiraishi

**Affiliations:** 1 Division of Anesthesiology, Niigata University Graduate School of Medical and Dental Sciences, 1-757, Asahimachi-dori, Chuo ward, Niigata, 951-8510 Japan; 2 Division of Thoracic and Cardiovascular Surgery, Niigata University Graduate School of Medical and Dental Sciences, 1-757, Asahimachi-dori, Chuo ward, Niigata, 951-8510, Japan

**Keywords:** omphalocele, pediatric cardiac surgery, upper abdominal drain-related pain, rectus sheath block, abdominal wall anatomy

## Abstract

**Background:**

Previous abdominal wall repair may alter the rectus abdominis muscle structure and render rectus sheath block (RSB) infeasible in pediatric cardiac surgery.

**Case Presentation:**

A 5-year-old boy with a history of siloplasty and omphalocele repair underwent right ventricle-to-pulmonary artery conduit replacement. Deep parasternal intercostal plane block and RSB had been planned. However, ultrasound pre-scanning failed to identify the rectus abdominis muscle in the upper abdomen, and the liver was visualized immediately beneath the abdominal wall. Preoperative computed tomography confirmed absence of the rectus abdominis muscle, rendering RSB anatomically infeasible. An alternative upper abdominal wall block was performed. The patient was extubated 2 h after admission to the intensive care unit and had no apparent discomfort from the chest and mediastinal drains.

**Conclusions:**

Preprocedural ultrasound assessment can identify altered rectus abdominis muscle structure and help anesthesiologists select a safer and potentially effective alternative analgesic strategy when RSB is not feasible.

## Background

Fast Track Surgery [1] or Enhanced Recovery After Surgery [2] concepts are increasingly being applied to pediatric cardiac surgery, and effective postoperative analgesia is an essential component of this approach. The efficacy of multisegmental intercostal nerve blocks for cardiac surgery with a median sternotomy has been reported in both adults [3, 4] and pediatric patients [5]. Furthermore, we proposed a perioperative anesthetic management method that combines deep parasternal intercostal plane block (PIPB), previously known as “transverse thoracic plane block” [6], and rectus sheath block (RSB) in pediatric cardiac surgery to relieve not only pain from the median sternotomy wound but also pain caused by a mediastinal drain inserted through the upper abdomen, and reported that this approach was associated with reduced intraoperative opioid requirements [7]. In fact, there have been many reports that the combined use of parasternal blocks such as deep PIPB and superficial PIPB, previously known as “pectointercostal fascial block” [6], with RSB contributes to reduced perioperative opioid use and improved postoperative pain scores [8–10].

Postoperative abdominal wall deformity and rectus abdominis diastasis after omphalocele repair have also been reported [11, 12]. However, to our knowledge, no previous reports have specifically described altered rectus anatomy affecting the feasibility of RSB and prompting a change in regional anesthesia strategy. Effective upper abdominal analgesia nevertheless remains necessary in such patients because mediastinal drains can cause substantial postoperative discomfort, even when RSB is anatomically infeasible. In patients with previous abdominal wall reconstruction, the rectus sheath may not be anatomically intact, and proceeding directly to RSB without confirming the anatomy may result in an ineffective or potentially unsafe peripheral nerve block. This case highlights the importance of preprocedural ultrasound assessment before attempting RSB in patients with previous abdominal wall reconstruction. Written informed consent was obtained from the parents of the patient for the publication of this report, including the figures and video.

## Case presentation

**Fig. 1:**
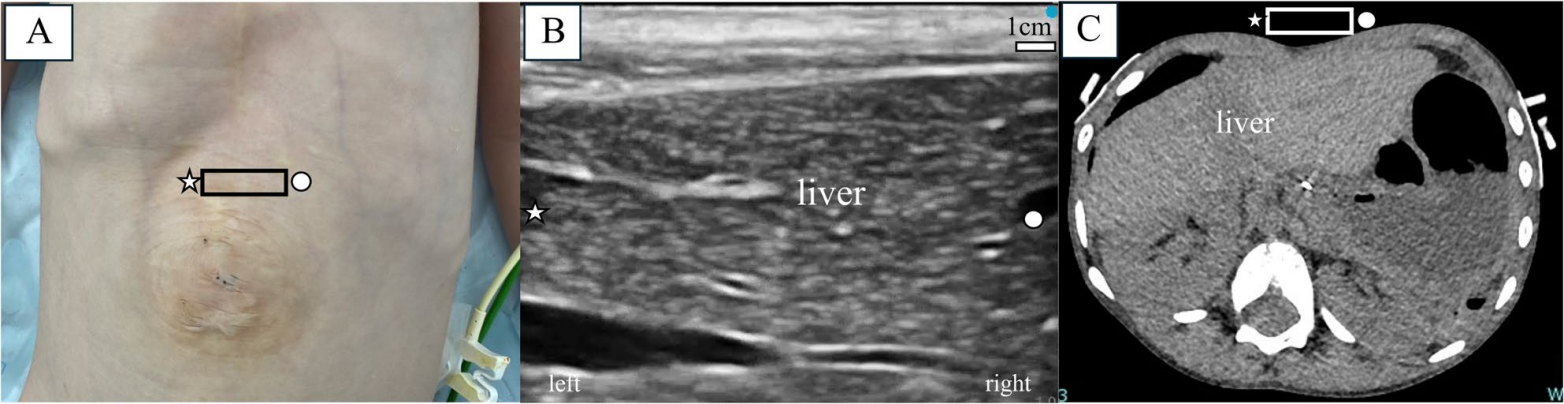
Ultrasound pre-scanning of the abdomen demonstrating absence of identifiable rectus abdominis muscle and preoperative computed tomography (CT) image at the same level. Rectangles indicate the position of the ultrasound probe. White stars and circles indicate the correspondence between the ultrasound probe and the orientation in the ultrasound and CT images. **A **The abdomen of the patient who had a siloplasty followed by an omphalocele repair as a newborn. The rectangle indicates the position of the linear ultrasound probe. **B** The preprocedural abdominal ultrasound scan did not identify the rectus abdominis muscle, and the liver lay immediately beneath the abdominal wall. **C** The absence of the rectus abdominis muscle was confirmed on the preoperative CT image at the same level

**Fig. 2:**
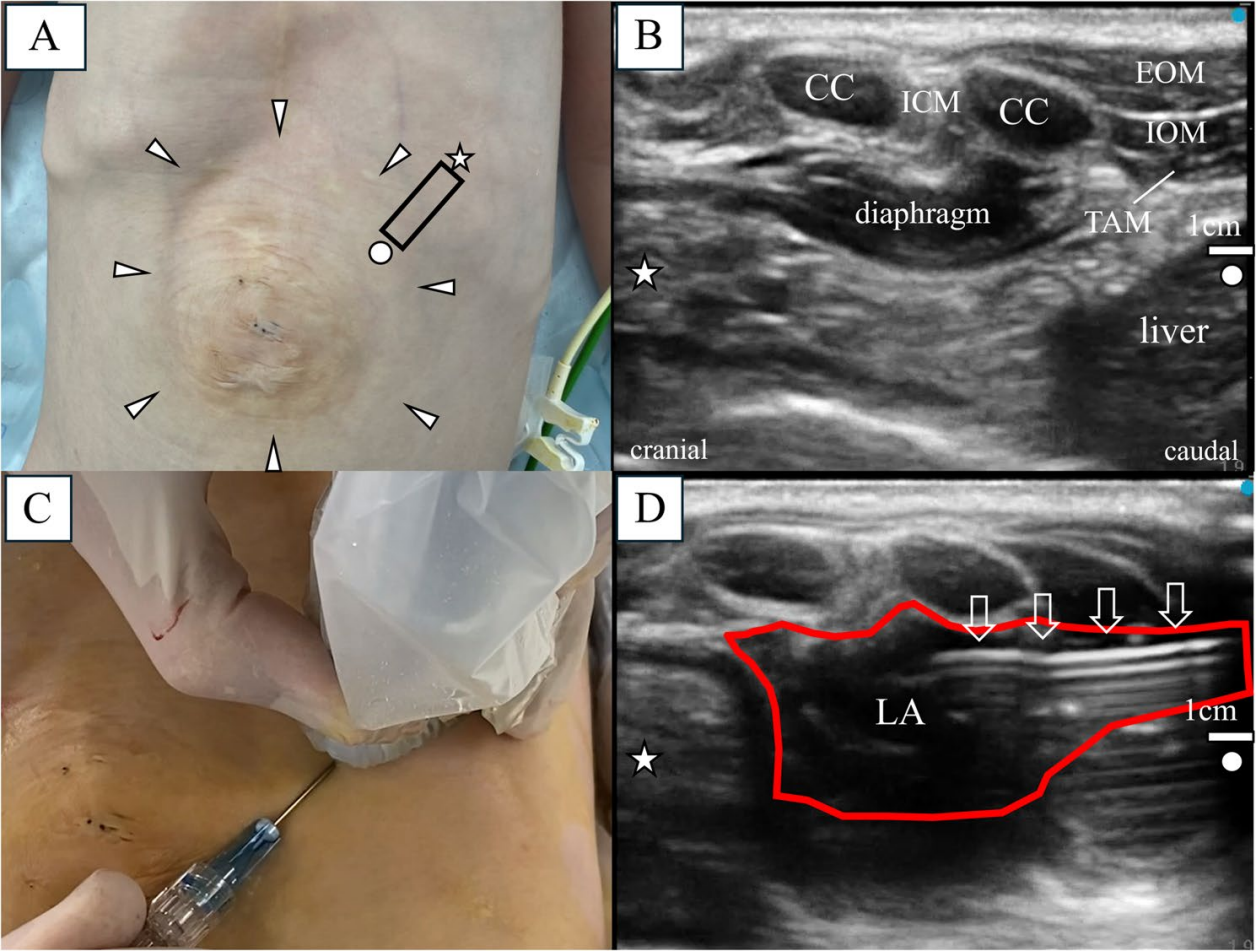
Ultrasound-guided upper abdominal wall block performed as an alternative to rectus sheath block. Because the rectus abdominis muscle structure was absent, an alternative upper abdominal wall block was performed instead of rectus sheath block. Rectangles indicate the position of the ultrasound probe. White stars and circles indicate the correspondence between the ultrasound probe and the orientation in the ultrasound images. **A** and **B** While checking the three-layer structure of the transverse abdominis, internal oblique, and external oblique muscles, the linear ultrasound probe was slid to the position where the internal oblique muscle was attached to the costal arch. The white triangles and rectangle indicate the area where the rectus abdominis muscle was absent after omphalocele repair and the position of the linear ultrasound probe, respectively. **C** and **D** 10 mL of 0.13% ropivacaine was injected into each side of the layer between the internal oblique and transverse abdominis muscles. This total dose was 1.68 mg/kg ropivacaine, which is below the maximum recommended dose for a pediatric patient in this case. The local anesthetic spread beyond the back of the ribs to the cranial side of the costal arch, pushing down the diaphragm [13, 14]. The arrow and red box indicate the needle and the spread of the administered local anesthetic, respectively. CC: costal cartilage, ICM: intercostal muscle, EOM: external oblique muscle, IOM: internal oblique muscle, TAM: transverse abdominis muscle, LA: local anesthetic. Video Caption. Video: Ultrasound-guided upper abdominal wall block performed as an alternative to rectus sheath block. Because the rectus abdominis muscle was not identifiable, an alternative upper abdominal wall block was performed instead of rectus sheath block. While checking the three-layer structure of the transverse abdominis, internal oblique, and external oblique muscles, the linear ultrasound probe was positioned where the internal oblique muscle was attached to the costal arch. The needle was inserted via a long-axis in-plane approach between the internal oblique and transversus abdominis muscles, and 10 mL of 0.13% ropivacaine was injected into each side. This total dose was 1.68 mg/kg ropivacaine, which is below the maximum recommended dose for a pediatric patient in this case. The local anesthetic spread beyond the back of the ribs to the cranial side of the costal arch, pushing down on the diaphragm

The patient was a 5-year-old male, 106 cm tall and weighing 15.5 kg. He was delivered via a cesarean section at 38 weeks because of suspected fetal liver prolapse. After birth, he was diagnosed with an omphalocele. Siloplasty was performed for the omphalocele, followed by omphalocele repair. He had a double outlet right ventricle and pulmonary stenosis, and underwent a modified Blalock-Taussig shunt procedure at 3 months of age, and a Rastelli procedure at 2 years of age. As he grew, relative stenosis of the Rastelli conduit progressed; therefore, the right ventricle-pulmonary artery conduit replacement was scheduled. Fast-track perioperative management using a combination of deep PIPB and RSB was planned, as is routinely performed at our institution. General anesthesia was induced by slow induction with sevoflurane and nitrous oxide. After securing a peripheral intravenous line, 10 mg of rocuronium and 100 µg of fentanyl were administered intravenously, and tracheal intubation was performed. General anesthesia was then maintained with sevoflurane and continuous intravenous administration of remifentanil until the end of surgery. After inserting a transesophageal echocardiogram, a central venous catheter was inserted. After that, a preprocedural ultrasound scan was performed before performing deep PIPB and RSB as usual. However, the preprocedural abdominal ultrasound scan did not identify the rectus abdominis muscle, and the liver was visualized immediately beneath the abdominal wall (Figs. 1A and B). The preoperative computed tomography image at the same level was checked again, and the absence of the rectus abdominis muscle was confirmed (Fig. 1C). Therefore, RSB was considered anatomically infeasible. After performing deep PIPB for the median sternotomy, we proceeded with an upper abdominal wall block along the costal margin using a perichondrial approach, with technical overlap with a modified thoracoabdominal nerve block through the perichondrial approach (m-TAPA) [13, 14] and subcostal transversus abdominis plane block (TAPB) [15–17], as an alternative to RSB to provide upper abdominal analgesia for mediastinal drain-related pain. While checking the three-layer structure of the transverse abdominis, internal oblique, and external oblique muscles, the linear ultrasound probe was slid to the position where the internal oblique muscle was attached to the costal arch (Figs. 2A and B, Video). Then, 10 mL of 0.13% ropivacaine was injected into each side (total 1.68 mg/kg ropivacaine) into the layer between the internal oblique and transverse abdominis muscles. The local anesthetic spread beyond the back of the ribs to the cranial side of the costal arch, pushing down the diaphragm (Figs. 2C and D, Video).

An additional 200 µg of fentanyl was administered at the start of surgery. The surgery, including cardiopulmonary bypass (CPB), was uneventful. After weaning from CPB and completion of modified ultrafiltration, continuous infusions of fentanyl, midazolam, and dexmedetomidine were initiated at 0.5 µg/kg/h, 0.1 mg/kg/h, and 1.0 µg/kg/h, respectively. The operation duration and CPB duration were 346 and 164 min, respectively. The patient was extubated approximately 2 h after admission to the intensive care unit. He did not report pain, and his hemodynamics remained stable after the extubation, despite receiving the above-mentioned sedatives. Continuous fentanyl infusion was discontinued on postoperative day (POD) 1. Dexmedetomidine and midazolam infusions were subsequently tapered, with dexmedetomidine discontinued on POD 2 and midazolam discontinued on POD 3 at the time of discharge from the intensive care unit. Bilateral chest drains were removed on POD 2, and the mediastinal drain was removed on POD 3. Although precise pain assessment using standard self-report tools, such as a visual analog scale, was not feasible owing to his age and mild intellectual disability, he remained capable of simple verbal communication. On repeated questioning, he consistently denied pain, and no rescue opioid analgesia was required during the postoperative course. Scheduled intravenous acetaminophen was also administered postoperatively.

## Discussion

This case highlights the clinical importance of preprocedural ultrasound assessment before attempting RSB in pediatric patients with previous abdominal wall reconstruction. The central message is not simply that an alternative block was performed, but that ultrasound pre-scanning revealed anatomy unsuitable for the planned RSB and prompted a safer and potentially effective alternative analgesic strategy. Furthermore, even the original TAPA block affects the anterior and lateral branches of the thoracoabdominal nerve and also covers sensation in the dermatomes from T5 to T12, making it suitable for abdominal surgery as well as chest and breast surgery [14]. It has been reported that an analgesic effect in the upper abdominal region, similar to that of RSB, can be achieved by puncturing at a more medial and superior level, i.e., a position similar to that used for the subcostal TAPB [15–17], and by injecting a larger volume of local anesthetic [18–20]. From this perspective, review of the insertion point of the peripheral nerve block performed in this case for upper abdominal analgesia suggests that it may be closer to that of subcostal TAPB than to m-TAPA (Fig. 2C, Video). Alternatively, because the ribs, intercostal muscle, and diaphragm were visible in the ultrasound image at the time of puncture and the administered local anesthetic spread beyond the back of the ribs to the cranial side of the costal arch, pushing down the diaphragm (Fig. 2D, Video), it is possible that the local anesthetic reached the “space between the endothoracic fascia, diaphragm, and costodiaphragmatic recess: SEDIC“ [21, 22]. Considering this, it is not inconsistent with the fact that satisfactory analgesia was achieved in the upper abdominal region in this case [23]. Compared with local anesthetic wound infiltration around the drain insertion site, an upper abdominal wall block may provide broader analgesic coverage, although wound infiltration is simpler and remains a reasonable alternative. Therefore, the present case should not be interpreted as proving that a peripheral nerve block is always necessary.

Although the precise classification of the peripheral nerve block performed in this case may overlap with techniques such as subcostal TAPB and SEDIC block, the present case suggests that alternative upper abdominal wall blocks can provide effective postoperative analgesia similar to that of RSB when the rectus abdominis muscle is absent or markedly altered. At the same time, because deep PIPB, systemic opioids, and postoperative sedatives were also administered, this report should not be interpreted as evidence of the independent efficacy of an alternative peripheral nerve block for mediastinal drain-related pain. Rather, it shows that preprocedural ultrasound pre-scanning can detect absence of the rectus abdominis muscle, prevent attempted peripheral nerve block placement into abnormal anatomy, and support timely selection of a safer and potentially effective alternative analgesic strategy for mediastinal drain-related pain in pediatric cardiac surgery. In patients with previous omphalocele repair, preprocedural ultrasound assessment of the upper abdominal wall may therefore be as important as the choice of peripheral nerve block itself. Familiarity with multiple peripheral nerve block techniques, rather than reliance on a single Plan A approach, is essential for tailoring appropriate analgesic strategies to patients with atypical abdominal wall anatomy.

## Data Availability

The data in this paper are available from the corresponding author on reasonable request.

## Abbreviations

CPB: cardiopulmonary bypass
CT: computed tomography
POD: postoperative day
PIPB: deep parasternal intercostal plane block
m-TAPA: modified thoracoabdominal nerve block through the perichondrial approach
RSB: rectus sheath block
TAPB: transversus abdominis plane block
